# No Decrease in Blood Pressure After an Acute Bout of Intermittent Hyperpnea and Hypoxia in Prehypertensive Elderly

**DOI:** 10.3389/fphys.2020.556220

**Published:** 2020-10-02

**Authors:** Jan Stutz, Ruben Oliveras, Remo Eiholzer, Christina M. Spengler

**Affiliations:** Exercise Physiology Lab, Institute of Human Movement Sciences and Sport, ETH Zürich, Zurich, Switzerland

**Keywords:** hyperpnea, respiratory muscle training, ventilation, hypoxia, blood pressure, elderly, sleep, hypertension

## Abstract

Prevalence of hypertension, subjective sleep complaints and snoring increases with age. Worse sleep and snoring, in turn, are independent risk factors to develop hypertension. Both respiratory muscle training (RMT) and intermittent hypoxia (IH) are suggested to have positive effects on these physiological and behavioral variables. This study therefore aimed to test the acute effects of a single bout of RMT, with and without IH, on resting blood pressure (BP) and sleep. Fourteen prehypertensive elderly performed a 60-min session of (a) intermittent voluntary normocapnic hyperpnea (HYP) alone, (b) HYP in combination with IH (HYP&IH) and (c) a sham intervention in randomized order. BP, hemodynamics, heart rate variability (HRV), cardiac baroreflex sensitivity (BRS) and pulse wave velocity (PWV) were assessed before and 15, 30 and 45 min after each intervention. Variables of sleep were assessed with actigraphy, pulse oximetry and with questionnaires during and after the night following each intervention. Neither HYP nor HYP&IH resulted in a decrease in BP. Repeated measures ANOVA revealed no significant interaction effect for systolic BP (*p* = 0.090), diastolic BP (*p* = 0.151), HRV parameters, BRS and PWV (all *p* > 0.095). Fragmentation index was lower after both HYP (−6.5 units) and HYP&IH (−8.4 units) compared to sham, *p*(ANOVA) = 0.046, although pairwise comparisons reveal no significant differences. There were no other significant effects for the remaining sleep variables. We conclude that one bout of intermittent hyperpnea, alone or in combination with IH, is not effective in lowering blood pressure or improving sleep in prehypertensive elderly.

## Introduction

Hypertension is a global public health issue, with aging being a major risk factor for the development of high blood pressure (World Health Organization, 2013). In the ≥ 65 year old Swiss population, hypertension affects about one out of two citizens (Federal Statistical Office, 2018), which poses substantial challenges to the healthcare system. Another healthcare challenge in the elderly is the high prevalence of subjective sleep complaints and snoring, both affecting > 50% of subjects aged ≥ 65 years (Foley et al., 1995; Stoohs et al., 1998). More importantly, evidence from epidemiologic and experimental studies suggest a higher risk for developing hypertension in subjects with inadequate sleep duration (Covassin and Singh, 2016) and in snorers (Khazaie et al., 2018); a single night of partial sleep deprivation has been shown to increase blood pressure (BP) and heart rate during the day (Lusardi et al., 1999). Therefore, safe and efficient strategies to improve BP and sleep in the hypertensive or pre-hypertensive elderly are needed, ideally with the capacity to improve BP and sleep within a very short time-frame.

While physical exercise leads to significant drops in blood pressure in the minutes to hours following activity – a phenomenon called post-exercise hypotension (PEH) (Thompson et al., 2001) – it might not be suitable for individuals who are unwilling or physically unable to engage in traditional activities such as resistance training, walking or cycling. Two alternative interventions with the potential to acutely lower BP and improve sleep without requiring activity from the upper or lower limbs are voluntary normocapnic hyperpnea (HYP) and intermittent hypoxia (IH). HYP can be seen as a form of exercise as it involves repetitive contractions of (upper body) skeletal muscles and shows typical cardiorespiratory responses to exercise as other modalities, such as an increase in heart rate, stroke volume and mean arterial pressure (Rodrigues et al., 2020). Previous research has shown that periods of HYP training, with sessions typically lasting 30 min while breathing at ∼60% of maximal voluntary ventilation, is successful in increasing the endurance of the respiratory muscles as well as whole-body exercise performance (Illi et al., 2012). While the muscle mass of the respiratory muscles is much smaller than that of the lower limbs, breathing at near-maximal capacity can result in O_2_ consumption by the respiratory muscles that accounts for up to 10–15% of whole-body maximal oxygen consumption (V̇O_2*max*_), so that overall intensity might be upwards of 20% V̇O_2max_ depending on fitness. As acute reductions in BP have already been observed following exercises with small muscle mass, such as the upper limbs (MacDonald et al., 2000) and with low intensity exercise, i.e., cycling at 30% V̇O_2max_ (Pescatello et al., 1991; Forjaz et al., 1998), we speculate that HYP could also lead to an acute drop in BP. From a mechanistic point of view, HYP might lower BP similarly to aerobic exercise, namely through changes in autonomic and/or vascular function (MacDonald et al., 1999; Chobanian et al., 2003), secondary to adaptations in the central baroreflex pathway and/or an increase in shear stress. While aerobic exercise typically results in a large increase in cardiac output, shear stress and consequent adaptations in vascular function (Schmidt et al., 2011), HYP is more likely to affect BP via the central baroreflex pathway and autonomic adaptations given the large BP swings seen during HYP (unpublished data). This is probably the result of the missing vasodilatory effect of the large muscles and the intrathoracic pressure swings directly affecting arterial pressure. IH, on the other hand, has long been considered a factor contributing to increases in BP, a view that stems from the findings in patients with obstructive sleep apnea, where severe hypoxia increases sympathetic discharge (Mateika et al., 2015). More recently, however, it has been suggested that systematic bouts of low/moderate hypoxia might actually contribute to lowering BP (Serebrovskaya et al., 2008), possibly via higher parasympathetic activation and/or hypoxic stimulation of endothelial nitric oxide production, with resulting vasodilation.

Sleep may also be improved in response to HYP and IH. On the one hand, HYP could be hypothesized to improve sleep through mechanisms similar to traditional forms of exercise, as exercise intensity does not seem to modulate the association between acute exercise and better sleep (Kredlow et al., 2015). On the other hand, HYP has the potential to improve sleep in elderly by reducing snoring, secondary to an increase in upper airway muscle activity, as observed after one session of inspiratory muscle training (How et al., 2007). This effect could be further amplified by the addition of IH: a single bout of IH has, in fact, been shown to enhance hypoglossal nerve and genioglossus muscle activity in humans and animals, potentially increasing airway patency (Mateika et al., 2015).

While there are reasons to believe that both HYP and IH could act to acutely improve BP and sleep in a cohort of pre-hypertensive elderly individuals, and that a combination of the two could further potentiate these effects, this possibility remains to be tested. Therefore, the primary aim of the present study was to investigate the acute effects of a single session of HYP, with and without concomitant IH, on BP in elderly with above normal resting BP [for safety and feasibility reasons BP was limited to systolic between 120–139 mmHg and/or diastolic BP between 80–89 mmHg, i.e., prehypertensive according to the seventh report of the Joint National Committee on high blood pressure (Chobanian et al., 2003)]. We hypothesized that one session of HYP would lead to an acute drop in BP and that the addition of IH would further potentiate this effect. Additionally, we hypothesized that one session of HYP would improve sleep and that the addition of IH would lead to larger effects compared to HYP alone.

## Materials and Methods

### Participants

Fourteen prehypertensive but otherwise healthy, non-smoking elderly completed the study (9m/5f; age, 69.2 ± 3.2 years; body mass index, 23.9 ± 2.0 kg⋅m^−2^; systolic BP, 133.9 ± 4.9 mmHg; diastolic BP 81.9 ± 5.7 mmHg; vital capacity, 115 ± 10%predicted; forced vital capacity, 109 ± 9%predicted; forced expiratory volume in 1 s, 106 ± 10%predicted; peak expiratory flow, 106 ± 19%predicted; peak inspiratory flow, 143 ± 21%predicted; maximal voluntary ventilation, 128 ± 24%predicted; maximal inspiratory pressure, 147 ± 40%predicted; maximal expiratory pressure, 106 ± 24%predicted; handgrip strength, 119 ± 13%predicted). Subjects refrained from caffeinated and alcoholic beverages before testing on test days, from intense exercise 48 h prior to testing, from any exercise 24 h prior to testing, and slept at least 7 h the two nights before test days. All participants signed the written informed consent form prior to any data collection. The study was approved by the Cantonal Ethics Committee of Zurich (Project ID 2017-00880) and registered on clinicaltrials.gov (NCT03313284). Power analysis was performed assuming a difference between means of 5 mmHg in systolic BP (Cohen Dz = 0.75 for a within participant design), power of 0.8 and an alfa level of 0.05. 5 mmHg was considered a meaningful and possible difference to be expected either between control and HYP, or between HYP and HYP&IH should an additive effect be present. The resulting sample size was 16 individuals. Two participants were excluded from the final analysis due to BP falling outside the pre-determined range at baseline. Inclusion of these participants in the final analysis did not change the presented results.

### Study Protocol

**FIGURE 1:**
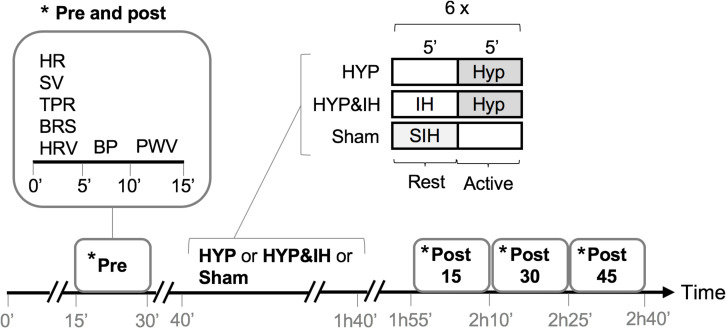
Study protocol of visits 2-4. BP, blood pressure; BRS, cardiac baroreflex sensitivity; HR, heart rate; HRV, heart rate variability; HYP, voluntary normocapnic hyperpnea; IH, intermittent hypoxia; PVW, pulse wave velocity; SIH, sham intermittent hypoxia; SV, stroke volume, TPR, total peripheral resistance.

A randomized crossover design was used to compare the acute effects of two different RMT interventions (intermittent HYP alone and in combination with IH) to a sham intervention. Participants reported to the laboratory on four different occasions, each separated by at least 48 hours. To avoid the influence of circadian variations of BP, all visits were scheduled at 1 pm. On the first visit, subjective sleep quality, daytime sleepiness, resting BP (after 10 min lying supine), lung function, respiratory muscle strength and handgrip strength were assessed, followed by a familiarization trial of HYP and IH. Before leaving the laboratory, participants were given a wrist-worn actigraph, a pulse oximeter and a hardcopy of the sleep questionnaire for sleep assessments at home. Finally, a randomization procedure determined the sequence of the three interventions (including sham) to be tested at visits 2-4. During visits 2-4, BP and related cardiovascular variables were assessed before and after a 60-min intervention (see Figure 1). Briefly, following a 15-min subject preparation phase (of which the last 5 min were in complete quiescence), baseline BP, hemodynamics, autonomic balance, baroreceptor function and pulse wave velocity were assessed (pre). The same variables were reassessed 15, 30 and 45 min after the end of the intervention (post 15, post 30 and post 45). During all measurements, subjects were lying supine on a stretcher and refrained from talking and moving. In addition to being prehypertensive at recruitment, the prehypertensive state (systolic BP between 120–139 mmHg and/or diastolic BP between 80–89 mmHg) was reassessed before each of the three interventions. If blood pressure was below or above these limits, the visit was rescheduled.

### Interventions

**FIGURE 2:**
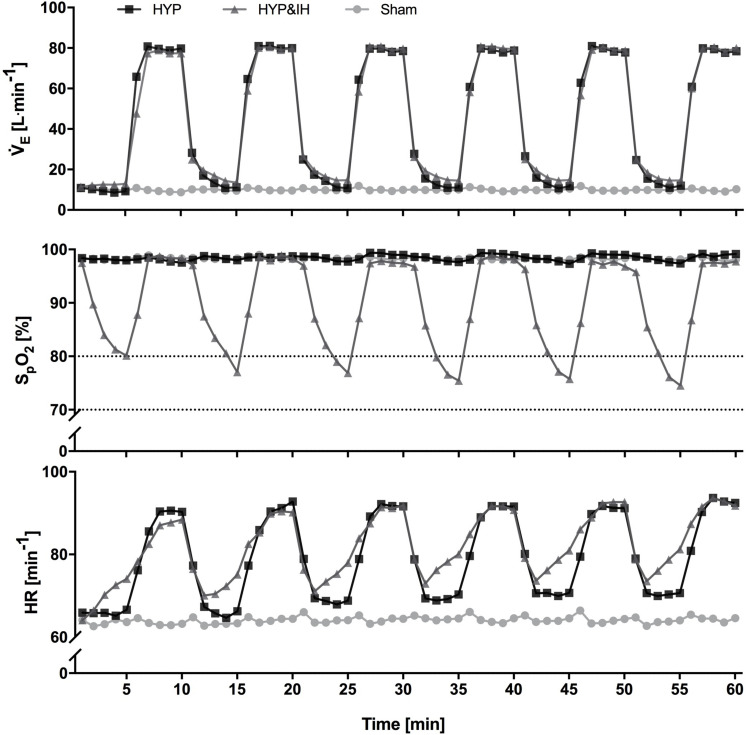
Time course of minute ventilation, peripheral oxygen saturation and heart rate (all *N* = 14) during one session of voluntary normocapnic hyperpnea (HYP), HYP with intermittent hypoxia (HYP&IH) and Sham. Statistical analyses are shown in Table 1. HR, heart rate; S_*p*_O_2_, peripheral oxygen saturation, V̇_*E*_, minute ventilation.

The time course within HYP and HYP&IH interventions are given in Figure 2. During each 60-min intervention, participants were sitting on a chair without backrest with knee and hip angles at approximately 90°.

#### Volitional Normocapnic Hyperpnea (HYP)

Hyperpnea consisted of six 5-min bouts of HYP with partial rebreathing of expired air using a commercially available device (SpiroTiger^®^, Idiag, Fehraltorf, Switzerland). Target ventilation was set at 60% of the individual maximal voluntary ventilation (MVV) assessed at visit 1. Tidal volume [50–60% of vital capacity (VC)] and breathing frequency (calculated accordingly) were held constant with the feedback of the device. In the 5-min breaks between HYP bouts, participants were breathing room air.

#### Volitional Normocapnic Hyperpnea With Intermittent Hypoxia (HYP&IH)

Hyperpnea with intermittent hypoxia also consisted of six 5-min bouts of HYP, similar to those described above. In the 5-min breaks, however, participants were exposed to normobaric hypoxia using a commercially available device (AltiTrainer200^®^, SMTEC, Nyon, Switzerland). Fraction of inspired oxygen (F_*i*_O_2_) was initially set at 10.5%, and then adjusted such that subjects reached an S_*p*_O_2_ of 70–80% within each 5-min exposure.

#### Sham

Sham consisted of six bouts of 5 min of sham hypoxia with subjects being connected to the Altitrainer^®^ device. Target fraction of inspired oxygen was set at 20.9%. In the 5-min breaks in between the sham hypoxic bouts, participants were disconnected from the device and breathed room air. During the intervention, participants were allowed to read an atlas or a hidden object book (both provided by the investigators).

### Assessments

#### Lung Function, Respiratory Muscle Strength and Handgrip Strength

Lung function was assessed according to current guidelines (Miller et al., 2005) using a metabolic cart (Oxycon pro, Jaeger, Höchberg, Germany), while respiratory muscle strength was recorded with a respiratory pressure meter (MicroRPM, Micro Medical/CareFusion, Kent, United Kingdom). Participants performed at least three maximal inspiratory and expiratory maneuvers until (a) the two best measurements were within 5% and until (b) the best maneuver was not the last one. Comparison to predicted values was done using age and sex specific reference equations for lung volumes (Quanjer et al., 2012), flows (Quanjer et al., 1993; Garcia-Rio et al., 2004), maximal voluntary ventilation (Cherniack and Raber, 1972) and inspiratory and expiratory muscle strength (Enright et al., 1993). Handgrip strength was assessed with an analog handgrip dynamometer (Takei Scientific Instruments Ltd., Niigata, Japan). Participants were sitting on a chair without backrest with knee and hip angles at approximately 90° and with their forearm placed horizontally on a table with an elbow angle of approximately 90°. Participants performed three maximal contractions starting with the dominant hand and then alternating hands. The best of the six measurements was used for further analyses. Data was compared to norm values from a Swiss population (Werle et al., 2009). Handgrip strength was assessed in 9 participants only since this measurement was added shortly after the start of the study.

#### Sleep

Sleep was assessed each night following a laboratory visit (total 4 nights) using wrist-worn actigraphy (Actiwatch Score, Cambridge Neurotechnology, Cambridge, United Kingdom), pulse oximetry (WristOx_2_, Nonin medical, Plymouth Minnesota, United States) and a 10-cm visual analog scale assessing tiredness at bedtime, quality of sleep and recovery after waking up. Time in bed (TIB), sleep efficiency (SE, defined as time asleep relative to time in bed), sleep onset latency (SOL) and fragmentation index [FI, defined as the amount and distribution of wakefulness during the night (Mezick, 2013)] were calculated automatically from the Sleep Analysis software (Cambridge Neurotechnology, Cambridge, United Kingdom). Basal S_*p*_O_2_ (S_*p*_O_2_ during non-event times, with event defined as a drop in S_*p*_O_2_ by at least 4% for a minimum duration of 10 s), adjusted Index (number of events per hour) and average HR were calculated automatically from the nVsion software (Nonin medical, Plymouth Minnesota, United States). Sleep after visit 1 was not analyzed as this served to account for first night effects. Subjective sleep quality [Pittsburgh Sleep Quality Index (PSQI)] and daytime sleepiness [Epworth Sleepiness Scale (ESS)] were assessed at the first visit to the laboratory for screening purposes.

#### Blood Pressure

Systolic and diastolic BP were measured as triplicates with 30 s between two consecutive measurements (timed from complete deflation of the cuff to the start of the next inflation). Measurements were performed in supine position, on the left arm at the height of the heart, using an oscillometric BP monitoring device (Cardiocap^TM^/5, Datex-Ohmeda Inc, Madison, WI, United States) or in sitting position (during the 60-min interventions). As recommended by the National Health and Nutrition Examination Survey (NHANES) procedures manual, the mean of the second and third measurement was used for further analyses. During the interventions, BP was assessed in the third minute of each 5-min interval. Subjects were sitting with their left arm resting on a table in order for the cuff to be at heart level and to reduce movement artifacts.

#### Hemodynamics and Autonomic Balance

Stroke volume (SV), HR, CO, HR variability (HRV) were calculated from signals of a trans-thoracic electrical bioimpedance device (PhysioFlow^®^, Manatec Biomedical, Petit Ebersviller, France). Skin preparation, electrode placement and calibration were performed according to manufacturer instructions. SV, HR and CO were processed with a moving median filter with a window size of 30 and then averaged over 5-min intervals (except in 4% of cases where a shorter interval was used due to artifacts). TPR was calculated from BP and CO values using the formula TPR = MAP / CO ⋅ 80, where MAP was calculated from systolic and diastolic BP using the formula MAP = DBP + 1/3 ⋅ (SBP - DBP). HRV was determined over 5-min intervals using the electrocardiogram (ECG) signal from Physioflow^®^. Data was recorded at 1 kHz and analyzed using Powerlab and Labchart Software v.8 (ADInstruments Ltd, Oxford, United Kingdom). Respiratory rate was not controlled during HRV assessment. Analysis was performed using the following settings: RR-interval between 800–1500 ms, complexity between 1–1.5, very low frequency (VLF) spectrum between 0–0.04 Hz, low frequency (LF) between 0.04–0.15 Hz and high frequency (HF) between 0.15–0.45 Hz. For three participants, the RR-interval range had to be widened based on visual inspection due to large variability and low resting HR. No data was removed from the analysis. The following parameters were chosen for further analyses: Standard deviation of the inter-beat-interval of normal sinus beats (SDNN) as a measure of overall variability, the root mean square of successive differences between normal heartbeats (RMSSD) as the time-domain measure to estimate parasympathetic activity, the high frequency (HF) band as the frequency-domain measure to estimate parasympathetic activity, the low frequency (LF) band as a marker of baroreflex activity and the LF/HF ratio as a measure of sympatho-vagal balance (Shaffer and Ginsberg, 2017). However, interpretation of HRV metrics should be views with caution, especially with respect to frequency domain analyses (Eckberg, 1997).

*Cardiac baroreflex sensitivity. Cardiac* baroreflex sensitivity (BRS) was assessed using continuous BP monitoring via a plethysmographic finger cuff (Nexfin, Edwards Lifesciences, Amsterdam, Netherlands). Inter-beat-Intervals (IBI) and corresponding BP values were analyzed using CardioSeries Software version 2.4. An up-sequence was defined as a series (≥ 3) of increase in systolic BP (≥ 1 mmHg) followed by a lengthening of the IBI (≥ 6 ms) of the subsequent heartbeat. A down-sequence was defined using the same criteria as for an up-sequence, but using decreases in systolic BP and shortenings of IBI. If there were no up or down sequences within the 5-min interval (4% of the cases), a lower BP threshold was used (0.2 mmHg instead of 1.0). All up-sequences and down-sequences within the 5-min periods were averaged and denominated BRS+ (average of up-sequences) and BRS- (average of down-sequences).

#### Pulse Wave Velocity

Carotid-femoral pulse wave velocity (PWV_*CF*_) was measured using a non-invasive device (Complior, Alam Medical, Vincennes, France). Measurements were performed as triplicates at each timepoint. If the three measurements were within 0.5 m⋅s^−1^ and quality above 90%, the values were averaged for further analyses. If not, the average of two values (19% of cases) or one single value (23% of cases) was used.

#### Ventilation, Gas Exchange, Peripheral Oxygen Saturation and Heart Rate

During the 60-min interventions, ventilation and gas exchange were measured continuously using a metabolic cart (Oxycon pro, Jaeger, Höchberg, Germany) while S_*p*_O_2_ and HR were assessed with pulse oximetry (Nellcor^TM^, Covidien, Minneapolis, United States). Data was recorded breath by breath and non-physiological values (i.e., zeroes, end-tidal CO_2_ values < 20 mmHg, S_*p*_O_2_ < 60% and loss of signal) were removed manually. Data was averaged over 1-min intervals for graphing purposes (Figure 2) and over 5-min intervals for data analysis (Table 1).

**TABLE 1:** Ventilatory and cardiovascular variables during volitional normocapnic hyperpnea (HYP), HYP with intermittent hypoxia (HYP&IH) and Sham.

		Rest	Active	ANOVA
		Mean ± SD	Mean ± SD	
V̇_*E*_ [L⋅min^–1^]	HYP	14.6 ± 4.3 ^$$^	76.1 ± 20.4 *** ^$$$^	*p* < 0.001
	HYP&IH	17.0 ± 5.0 ^$$$^	74.9 ± 20.8 *** ^$$$^	
	Sham	10.0 ± 2.6	9.9 ± 2.0	
f_*R*_ [min^–1^]	HYP	18.1 ± 3.4 ^$$^	32.4 ± 4.1 *** ^$$$^	*p* < 0.001
	HYP&IH	17.6 ± 4.1 ^$$$^	31.8 ± 4.2 *** ^$$$^	
	Sham	14.2 ± 2.8	15.7 ± 2.4	
V_*T*_ [L]	HYP	0.82 ± 0.26	2.28 ± 0.44 *** ^$$$^	*p* < 0.001
	HYP&IH	1.00 ± 0.31 ^$$^	2.26 ± 0.45 *** ^$$$^	
	Sham	0.74 ± 0.20	0.68 ± 0.19 ***	
F_*i*_O_2_ [%]	HYP	20.8 ± 0.1	20.5 ± 0.3 * ^$$^	*p* < 0.001
	HYP&IH	10.6 ± 0.6 ^$$$^^£££^	20.0 ± 0.2 *** ^$$$^^£££^	
	Sham	20.7 ± 0.2	20.9 ± 0.1	
S_*p*_O_2_ [%]	HYP	98.1 ± 1.4	98.7 ± 1.0	*p* < 0.001
	HYP&IH	84.1 ± 1.6 ^$$$^^£££^	95.8 ± 1.8 *** ^$$$^^£££^	
	Sham	98.2 ± 1.1	98.4 ± 1.1	
P_*ET*_CO_2_ [mmHg]	HYP	29.5 ± 2.9 ^$$^	29.9 ± 4.7	*p* = 0.001
	HYP&IH	28.7 ± 2.3 ^$$$^	29.3 ± 4.2	
	Sham	33.9 ± 2.6	32.9 ± 2.3 ***	
Heart Rate [min^–1^]	HYP	70.1 ± 11.4 ^$$^	88.4 ± 14.7 *** ^$$$^	*p* < 0.001
	HYP&IH	75.0 ± 13.6 ^$$$^^£^	88.9 ± 14.4 *** ^$$$^	
	Sham	64.0 ± 11.6	64.2 ± 11.0	
Systolic blood pressure [mmHg]	HYP	128.1 ± 5.6	174.3 ± 10.3 *** ^$$$^	*p* < 0.001
	HYP&IH	134.4 ± 10.9	177.8 ± 9.8 *** ^$$$^	
	Sham	128.8 ± 9.2	128.0 ± 9.2	
Diastolic blood pressure [mmHg]	HYP	80.1 ± 6.5	102.3 ± 12.3 *** ^$$$^	*p* < 0.001
	HYP&IH	83.2 ± 9.0	109.1 ± 8.8 *** ^$$$^	
	Sham	81.3 ± 6.9	80.6 ± 6.8	
Breathlessness [points]	HYP	0.8 ± 0.8	2.7 ± 2.8 * ^$$$^	*p* < 0.001^*a*^
	HYP&IH	1.9 ± 2.0	2.4 ± 2.3 ^$$$^	
	Sham	0.9 ± 1.1	0.7 ± 0.9	
Respiratory exertion [points]	HYP	0.9 ± 0.8	5.3 ± 2.7 *** ^$$$^	*p* < 0.001^*a*^
	HYP&IH	1.9 ± 1.8	4.3 ± 2.6 ^$$$^	
	Sham	0.9 ± 1.1	0.7 ± 0.9	

*Data are shown as 30-min averages. One subject excluded from blood pressure analysis due to inability to measure blood pressure during HYP. V̇_E_, minute ventilation; f_R_, breathing frequency; V_T_, tidal volume; F_i_O_2_, fraction of inspired O_2_; S_p_O_2_, peripheral oxygen saturation; P_ET_CO_2_, end-tidal partial pressure of CO_2_. ^a^ Friedman’s ANOVA. ^∗^p < 0.05, ^∗∗^p < 0.01 and ^∗∗∗^p < 0.001, within intervention (vs. Rest). ^$^ p < 0.05, ^$$^p < 0.01 and ^$$$^p < 0.001, between intervention (vs. Sham). ^£^ p < 0.05, ^££^p < 0.01 and ^£££^p < 0.001, between intervention (vs. HYP).*

#### Breathlessness and Respiratory Exertion

Perception of BR (the sensation of “not getting enough air”) and RE (“work/effort that is required by breathing”) were assessed during the 60-min intervention at the end of each 5-min interval with a 10-cm visual analog scale, labeled with “none” on the left end point and “maximal” at the right end point.

### Data Analysis

#### Interventions – Ventilation, Respiratory Sensations, Oximetry, Heart Rate and Blood Pressure

For each 60-min intervention (HYP, HYP&IH and sham), two 30-min averages were calculated, namely (1) the average of the six 5-min active intervals and (2) the average of the six 5-min rest intervals (see Figure 1). Distribution of these averages was first checked for normality using the Kolmogorov–Smirnov test. If normality was violated, Friedman’s analysis of variance (ANOVA) was used, otherwise a one-way repeated measures ANOVA. If sphericity was violated, the Greenhouse-Geisser correction was applied. Significant effects were further investigated using Bonferroni-adjusted pairwise comparisons to perform within and between intervention comparisons (9 comparisons).

#### Blood Pressure and Cardiovascular Parameters

Blood pressure, HR, SV, CO, TPR, HRV, BRS and PWV_*CF*_ were analyzed with a two-way repeated measures ANOVA. Factors were *Intervention* with 3 levels (HYP, HYP&IH, sham) and *Time* with 4 levels (pre, post 15, post 30 and post 45). If sphericity was violated, the Greenhouse-Geisser correction was used. Post-hoc simple effect analyses were further used to evaluate the effect of *Time* for each intervention separately and the effect of *Intervention* for each timepoint separately (i.e. Bonferroni-adjusted pairwise comparisons).

#### Sleep

Whole night averages for actigraphy and oximetry data and subjective sleep quality were first checked for normality using the Kolmogorov–Smirnov test. If normality was violated, Friedman’s ANOVA was used. Otherwise, a one-way repeated measures ANOVA was used to compare sleep after the three interventions. If sphericity was violated, the Greenhouse-Geisser correction was used. Significant effects were further investigated using Bonferroni-adjusted pairwise comparisons (3 comparisons).

#### Correlation Analyses

Repeated measures correlations were done using an R package (rmcorr) provided by Bakdash and Marusich (Bakdash and Marusich, 2017). Correlations were done between BP and TPR for each intervention separately with the repeated measure variable being *Time* with 4 levels (pre, post 15, post 30 and post 45).

Significance was set at *p* ≤ 0.05 for all analyses. ANOVAs, simple effect analyses and Bonferroni-adjusted pairwise comparisons were performed using IBM SPSS Software version 25 (IBM Corp., Armonk, NY, United States). Repeated measures correlation was performed using R version 3.6.0 (R Core Team, Vienna, Austria).

## Results

### Interventions

Cardiovascular and ventilatory data assessed during the interventions are shown in Figure 2 and Table 1. In brief, systolic BP, diastolic BP, HR, ventilation, breathing rate and tidal volume were significantly higher during hyperpnea (i.e., active intervals) compared to rest intervals in both HYP and HYP&IH, with no difference between the two interventions. S_*p*_O_2_ was significantly lower during HYP&IH compared to Sham while it remained unchanged during HYP.

### Blood Pressure

**FIGURE 3:**
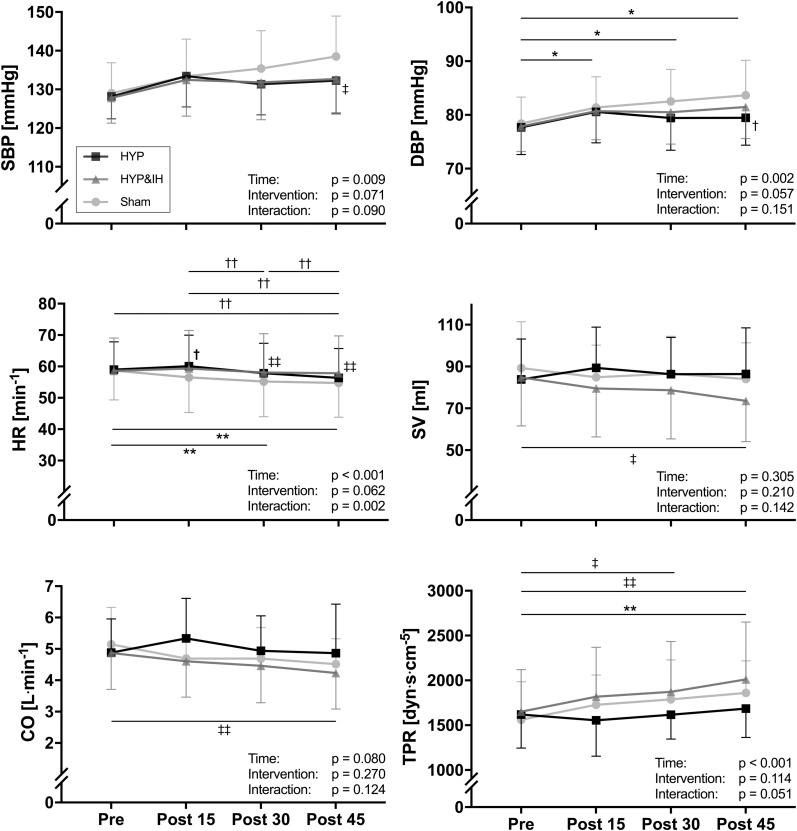
Blood pressure and hemodynamic parameters (all *N* = 14) before and after one session of voluntary normocapnic hyperpnea (HYP), HYP with intermittent hypoxia (HYP&IH) and Sham. CO, cardiac output; DBP, diastolic blood pressure; HR, heart rate; SBP, systolic blood pressure; SV, stroke volume; TPR, total peripheral resistance. *p < 0.05 and **p < 0.01, Sham, within intervention. ^†^*p* < 0.05 and ^††^*p* < 0.01, HYP, within or between intervention (vs. Sham). ^‡^*p* < 0.05 and ^‡‡^*p* < 0.01, HYP&IH, within or between intervention (vs. Sham).

Neither HYP nor HYP&IH resulted in lower BP in the 45 min post-exercise (Figure 3). For systolic BP, repeated measures ANOVA revealed a significant main effect of time, *F*(1.92, 25.01) = 5.79, *p* = 0.009; no main effect of intervention, *F*(2, 26) = 2.94, *p* = 0.071 and no interaction effect, *F*(6, 78) = 1.91, *p* = 0.090. Simple effects analyses revealed that systolic BP at post 45 was significantly higher after sham compared to HYP&IH (mean difference +5.8 mmHg, *p* = 0.035) but not compared to HYP (mean difference +6.3 mmHg, *p* = 0.094). After sham, systolic BP increased with time and almost reached statistical significance at post 45 vs pre (mean difference +9.5 mmHg, *p* = 0.051). There was no difference between HYP and HYP&IH at any timepoint (all *p* = 1.000).

For diastolic BP there was also a significant main effect of time, *F*(1.74, 22.59) = 8.77, *p* = 0.002; no main effect of intervention, *F*(2, 26) = 3.20, *p* = 0.057 and no interaction effect, *F*(6, 78) = 1.63, *p* = 0.151. Simple effects analyses revealed that diastolic BP at post 45 was significantly higher after Sham compared to HYP (mean difference +4.2 mmHg, *p* = 0.035). After sham, diastolic BP increased with time and was significantly higher at post 15, post 30 and post 45 compared to pre (all *p* < 0.050). There was no difference between HYP and HYP&IH at any timepoint (all *p* > 0.250).

### Stroke Volume, Heart Rate, Cardiac Output, Total Peripheral Resistance, Heart Rate Variability, Cardiac Baroreflex Sensitivity and Pulse Wave Velocity

A significant main effect of time was present for HR, *F*(1.86, 24.23) = 12.49, *p* < 0.001 and for TPR, *F*(3, 39) = 11.36, *p* < 0.001; no main effect of intervention for HR, *F*(2, 26) = 3.10, *p* = 0.062 nor for TPR, *F*(2, 26) = 2.36, *p* = 0.114; and a significant interaction effect for HR, *F*(2.77, 36.00) = 6.20, *p* = 0.002 but not for TPR, *F*(6, 78) = 2.20, *p* = 0.051. Significant simple effects are shown in Figure 3. In brief, HR decreased with time after Sham and HYP but not after HYP&IH while TPR increased after Sham and after HYP&IH but not after HYP. For SV and CO, there was no significant main effect of time, intervention, and no interaction effect (all *p* > 0.080). However, both SV and CO decreased with time after HYP&IH, reaching statistical significance at post 45 compared to pre (Figure 3). There was no other significant main effect of time, intervention, or interaction effect for HRV parameters, BRS or PWV_*CF*_ (all *p* > 0.095, Table 2).

**TABLE 2:** Cardiovascular variables before and after one session of voluntary normocapnic hyperpnea (HYP), HYP with intermittent hypoxia (HYP&IH) and Sham.

		Pre	Post 15	Post 30	Post 45	Time	Intervention	Interaction
		Mean ± SD	Mean ± SD	Mean ± SD	Mean ± SD			
SDNN [ms]	HYP	39.1 ± 20.4	46.1 ± 25.0	50.4 ± 25.1	52.3 ± 24.7	*p* = 0.117	*p* = 0.401	*p* = 0.095
	HYP&IH	46.8 ± 40.0	35.3 ± 16.7	45.6 ± 22.0	46.5 ± 21.7			
	Sham	40.5 ± 32.5	51.6 ± 24.2	52.3 ± 28.9	57.1 ± 31.4			
RMSSD [ms]	HYP	35.9 ± 28.9	45.0 ± 33.7	48.7 ± 30.6 ^†^	50.7 ± 38.1 ^†^	*p* = 0.168	*p* = 0.605	*p* = 0.203
	HYP&IH	43.0 ± 53.1	33.9 ± 29.6	43.3 ± 33.1 ^‡^	41.6 ± 34.4 ^‡^			
	Sham	38.4 ± 45.5	43.9 ± 27.0	51.0 ± 35.8	50.4 ± 40.6			
LF Power [%]	HYP	26.3 ± 13.3	25.1 ± 10.0	26.8 ± 8.9	29.7 ± 10.8	*p* = 0.803	*p* = 0.355	*p* = 0.792
	HYP&IH	25.7 ± 14.5	24.5 ± 9.2	21.5 ± 12.4	21.9 ± 9.1			
	Sham	26.3 ± 17.5	24.8 ± 8.3	24.2 ± 12.1	27.2 ± 12.1			
HF Power [%]	HYP	24.4 ± 17.6	26.0 ± 15.5	28.6 ± 18.0	31.2 ± 18.5	*p* = 0.704	*p* = 0.977	*p* = 0.100
	HYP&IH	24.5 ± 18.6	32.6 ± 22.2	29.8 ± 25.2	24.3 ± 18.8			
	Sham	30.8 ± 18.1	27.7 ± 17.4	30.9 ± 15.6	23.5 ± 14.0			
LF/HF	HYP	1.5 ± 1.1	1.3 ± 0.9	1.5 ± 1.4	1.4 ± 1.0	*p* = 0.706	*p* = 0.477	*p* = 0.258
	HYP&IH	2.1 ± 2.4	1.1 ± 0.7	1.7 ± 1.8	1.4 ± 1.2			
	Sham	1.2 ± 1.1	1.3 ± 0.8	0.9 ± 0.5	1.4 ± 0.7			
BRS+ [ms⋅mmHg^–1^]	HYP	8.9 ± 5.4	9.1 ± 4.3	10.8 ± 4.9	11.8 ± 6.1	*p* = 0.422	*p* = 0.875	*p* = 0.217
	HYP&IH	12.1 ± 11.8	7.5 ± 4.0 ^£^	10.0 ± 5.0	11.0 ± 7.3			
	Sham	10.1 ± 5.1	11.3 ± 4.2	10.7 ± 4.3	10.4 ± 5.3			
BRS- [ms⋅mmHg^–1^]	HYP	11.8 ± 5.0	12.6 ± 8.2	12.0 ± 6.4	11.2 ± 6.9	*p* = 0.929	*p* = 0.318	*p* = 0.653
	HYP&IH	11.0 ± 7.0	9.5 ± 4.3	10.7 ± 6.4	10.9 ± 6.1			
	Sham	11.5 ± 6.3	11.8 ± 7.9	12.2 ± 6.2	10.4 ± 6.2			
PWV_*CF*_ [m⋅s^–1^]	HYP	8.7 ± 2.8	8.8 ± 2.6	8.7 ± 2.9	8.6 ± 2.6	*p* = 0.103	*p* = 0.859	*p* = 0.238
	HYP&IH	8.3 ± 2.7	9.0 ± 2.8	9.5 ± 3.9	9.3 ± 3.4			
	Sham	8.7 ± 2.8	9.0 ± 2.9	9.2 ± 2.7	9.3 ± 2.8			

*Two subjects excluded from HRV analyses due to improper ECG signal, two subjects excluded from BRS analyses due to lack of up or down ramps within the 5-min intervals, and one subject excluded from PWV analysis due to inability to detect acceptable pulse wave signals. SDNN, standard deviation of the inter-beat-interval of normal sinus beats; RMSSD, root mean square of successive differences between normal heartbeats; LF, low frequency; HF, high frequency; BRS, cardiac baroreflex sensitivity; PWV_*CF*_, carotid femoral pulse wave velocity. ^†^*p* < 0.05, ^††^*p* < 0.01 and ^†††^*p* < 0.001, within intervention (vs Pre). ^‡^*p* < 0.05, ^‡‡^*p* < 0.01 and ^‡‡‡^*p* < 0.001, within intervention (vs Post 15). ^£^*p* < 0.05, ^££^*p* < 0.01 and ^£££^*p* < 0.001, between intervention (vs. Sham).*

### Repeated Measures Correlations

There was a significant positive correlation between systolic BP and TPR for sham (*r* = 0.42, *p* = 0.005) and for HYP&IH (*r* = 0.35, *p* = 0.021), but not for HYP (*r* = 0.06, *p* = 0.717). Diastolic BP was also correlated to TPR for sham (*r* = 0.43, *p* = 0.004) and for HYP&IH (*r* = 0.53, *p* < 0.001), but not for HYP (*r* = −0.06, *p* = 0.700).

#### Sleep

One-Way repeated measures ANOVA revealed a significant effect for FI, *F*(2, 24) = 3.50, *p* = 0.046. After both HYP and HYP&IH, FI was lower compared to Sham, although Bonferroni corrected pairwise comparisons were not significant (*p* > 0.115). There were no other significant effects for the remaining sleep variables (Table 3). Baseline PSQI and ESS score averaged 3.5 ± 2.1 and 6.2 ± 3.4 points, respectively.

**TABLE 3:** Objective and subjective sleep variables after one session of voluntary normocapnic hyperpnea (HYP), HYP with intermittent hypoxia (HYP&IH) and Sham.

	HYP	HYP&IH	Sham	ANOVA
		
	Mean ± SD	Mean ± SD	Mean ± SD	
Time in bed [min]	478 ± 48	471 ± 79	462 ± 69	*p* = 0.786
Sleep onset latency [min]	10.3 ± 13.9	8.0 ± 9.9	7.9 ± 8.0	*p* = 0.444^*a*^
Sleep efficiency [%]	86.3 ± 8.2	86.8 ± 7.5	83.9 ± 6.8	*p* = 0.317
Fragmentation Index	35.9 ± 17.9	34.0 ± 15.4	42.4 ± 14.8	*p* = 0.046
Basal S_*p*_O_2_ [%]	93.2 ± 1.3	93.1 ± 1.3	93.0 ± 1.2	*p* = 0.686
Adj. Index [Events⋅h^–1^]	5.5 ± 3.4	4.6 ± 2.6	5.4 ± 3.2	*p* = 0.189
Heart rate [min^–1^]	61 ± 9	61 ± 9	61 ± 10	*p* = 0.898
Tiredness [points]	4.9 ± 2.4	5.5 ± 2.7	4.5 ± 2.5	*p* = 0.215
Sleep quality [points]	6.9 ± 2.7	6.8 ± 2.6	7.0 ± 1.9	*p* = 0.980
Recovery [points]	7.8 ± 1.4	7.9 ± 1.4	7.7 ± 1.6	*p* = 0.904

*One subject excluded from actigraphy analysis due to low battery and one subject excluded from oximetry analysis due to forgetting to wear the device. Basal S_p_O_2_, peripheral oxygen saturation during non-event times. ^a^ Friedman’s ANOVA.*

## Discussion

This study is the first to evaluate the acute effects of intermittent HYP with and without IH on BP and sleep. The effects on BP were investigated for up to 45 min after the end of a 60-min intervention and sleep was recorded during the night after each intervention. The main findings are that a) an acute bout of HYP does not result in a drop in BP nor better sleep in prehypertensive elderly, and that b) the addition of IH to HYP does not yield additional positive effects.

### Hyperpnea and Blood Pressure

One session of HYP did not lower blood pressure in the 45 min after stimulus cessation. This is in contrast with the well-known PEH effect seen after one session of whole-body exercise, where systolic BP is reduced by 5–15 mmHg in prehypertensive subjects for up to several hours (MacDonald, 2002; Liu et al., 2012). Several arguments may explain the lack of changes in BP after HYP. First, in contrast to whole-body exercise, HYP targets only a relatively small muscle mass. This might be of relevance since exercise targeting a larger muscle mass is presumably associated with a greater vasodilation of blood vessels to the skin and to the active muscles after the end of exercise, both contributing to a larger decrease in TPR and therefore BP. However, MacDonald et al. (2000), comparing the effects of 30 min of leg with 30 min of arm ergometry exercise, found similar decreases in BP after both conditions, leading them to conclude that the exercising muscle mass does not affect the magnitude of PEH (MacDonald et al., 2000). It remains possible, however, that a threshold exists to how small a muscle mass is needed in order to lower BP post-exercise.

Second, relative intensity of HYP [i.e., 55% of predicted maximal HR, corresponding to very light intensity according to the American College of Sport Medicine criteria (Garber et al., 2011)], might be too low to lower BP post-exercise. Whether there is indeed a dose-response relationship between exercise intensity and magnitude of BP drop post-exercise is controversial as some studies showed a relationship (Hagberg et al., 1987) whereas others did not (Forjaz et al., 1998). Light intensity cycling for 30 min (low total work), for example, did not result in PEH, whereas 30 min of vigorous intensity and 50 min of light intensity cycling (with the same total work, expressed in W⋅s) resulted in PEH with no difference between the two conditions (Jones et al., 2007), suggesting that total work performed is a better indicator of whether a given activity can or not elicit subsequent drops in BP. Total work done during HYP in this study is presumably low given the very small muscle mass used and relatively low cardiovascular strain. Still, intensities as low as 30% of V̇O_2max_ have also been shown to result in PEH (Forjaz et al., 1998), as well as resistance exercise performed at only 40% of maximal capacity (Rezk et al., 2006) and therefore it is unlikely that lack of intensity alone could explain our results. Considering our participants as being of average fitness, aerobic capacity for their age group would be estimated at roughly 8.5 METS (Mandsager et al., 2018). The work performed by the respiratory muscles can account for 10–15% of whole-body VO_2_ at near maximal voluntary ventilation (Turner et al., 2012), and thus to 0.85 – 1.3 METS above rest. This would equate to 1.85 – 2.3 METS, or 23–27% of V̇O_2max_, not dissimilar to the lower range of intensities seen when whole-body exercise is employed.

Third, adaptations in the central baroreflex pathway, which seems to play an important role in mediating PEH after exercise (Chen and Bonham, 2010), might differ between exercise modalities, due to different BP responses during exercise. During HYP, for example, both systolic and diastolic BP are increased, whereas during aerobic exercise only the former is increased. On the other hand, the BP response during dynamic resistance training (Cavalcante et al., 2015) can be very similar to that seen in the current study. Since the magnitude of PEH seems to be independent of exercise modality [for example resistance vs aerobic (MacDonald, 2002)] and intensity [for example resistance training performed at 40% vs 80% of the 1-repetition maximum (Cavalcante et al., 2015)], we deduce that the lack of changes in BP after HYP cannot be traced back to differences in baroreceptor stimulation but must reside in peripheral factors.

### The Addition of Intermittent Hypoxia to Hyperpnea on Blood Pressure

The addition of IH to HYP did not have an effect on the BP response after exercise. From previous studies we hypothesized to find an altered autonomic balance towards increased parasympathetic activity after IH exposure. However, autonomic balance was unaltered not only after HYP but also after HYP&IH. Although we were aware that unaltered HRV parameters 10 min after one session of IH have already been reported (Chacaroun et al., 2016), we tested the possibility that a decreases in sympatho-vagal balance would manifest later into the recovery phase, which was not the case (at least when combined with HYP). We therefore suggest that, at least acutely, the addition of IH does not alter the autonomic response and BP more than one bout of intermittent HYP alone. Our results do, however, reinforce the finding that while it is believed that IH-mediated sympathetic activation is likely an important contributor to the development of hypertension in obstructive sleep apnea patients (Konecny et al., 2014), the autonomic response to IH seems to be dependent on the dose and duration of the IH stimuli (see Mateika et al., 2015 for a review).

Although neither HYP nor HYP&IH resulted in lower BP post-exercise, there was a trend for an interaction effect in systolic BP (*p* = 0.090) due to an increase in BP after Sham (+9.5 mmHg at post 45 vs. pre). This increase could be the result of circadian variations in BP (Mancia, 2012) and/or lying for a prolonged time on a stretcher with a consequent drop in body temperature (a few participants reported feeling cold during the post measurements after Sham). Theoretically, a drop in temperature leads to peripheral vasoconstriction, resulting in an increase in TPR and a consequent increase in BP. In our study, TPR was increased after Sham and this increase was significantly correlated with the increase in both systolic and diastolic BP, speaking in favor of this assumption. Finally, P_*ET*_CO_2_ was lower during the rest intervals of HYP and HYP&IH compared to Sham. However, if anything, lower P_*ET*_CO_2_ should bias the results towards reduced BP, and not the opposite (Burnum et al., 1954).

### Hyperpnea and Sleep

Based on baseline ESS and PSQI scores, participants of this study can be characterized as good sleepers (Johns, 1991; Smyth, 2008). This might explain the lack of effects of HYP on sleep (ceiling effects). On the other hand, an acute bout of whole-body exercise improves sleep in subjects without sleep complaints (Kredlow et al., 2015). Since participants of the present study reported being aerobically active, sleep quality might already be at a level where additional interventions cannot further contribute to positive adaptions.

Nonetheless, 5 participants out of 14 reported loud snoring at least once a week. As snoring has been found to be a determinant of lower SE and higher wake after sleep onset (Hoffstein et al., 1991), we performed a post hoc analysis on this subgroup, which showed signs of impaired sleep. FI and desaturation index, for example, were higher when compared to the non-snorer subgroup (+11.4 points, *p* < 0.05, and +5.2 events⋅h^−1^, *p* < 0.05, analyses not shown). More importantly, after HYP, FI decreased in the snoring subgroup (−12.8 points, *p* < 0.05) but not in the non-snorer subgroup (−3.1 points, *p* > 0.05). Also, SE increased and desaturation index decreased after HYP in the snorer subgroup, although failing to reach statistical significance. These data point towards possible positive adaptations after a single HYP session in snorers. However, this hypothesis remains to be tested in a larger group including only subjects that suffer from snoring and/or sleep disordered breathing.

While the acute effects of HYP had not yet been tested on sleep, other types of exercises involving the respiratory muscles have already been shown to have positive effects on sleep in patients. Myofunctional therapy training - consisting of isotonic and isometric exercises for oral and oropharyngeal structures – improves apnea-hypopnea index and snoring in obstructive sleep apnea patients, hypothetically through increased tone of upper airway dilator muscles (Camacho et al., 2015). Also, four weeks of respiratory muscle endurance training were shown to reduce snoring in healthy middle aged adults (Furrer-Boschung, 1997). Whether one session of HYP also results in increased upper airway dilator muscle activity, and whether this results in less snoring and increased SE and FI remains to be tested.

### The Addition of Intermittent Hypoxia to Hyperpnea on Sleep

The addition of IH to HYP did not have any additional effect on sleep. We hypothesized that IH would improve sleep since data from animal and human studies have shown that genioglossus muscle (an upper airway dilator muscle) activity is increased after a single exposure to IH, which could in turn increase airway patency and reduce upper airway resistance, especially in snorers and obstructive sleep apnea patients (Mateika et al., 2015). However, given the lack of effects in sleep outcomes after HYP&IH compared to HYP (in the whole group and in the snorer subgroup), we conclude that the small positive effects seen after the two interventions are attributable to HYP rather than the addition of IH.

### Limitations

Different factors limit the generalizability and validity of the current findings. First, we tested prehypertensive but otherwise healthy and active elderly, and cannot exclude the possibility that a drop in BP occurs in a hypertensive and/or sedentary population after one session of HYP. However, PEH after whole-body exercise occurs irrespective of baseline BP levels or activity status (MacDonald, 2002). Second, by testing only subjects with already good sleep, we cannot infer whether HYP improves sleep in subjects with subjective sleep complaints and whether this has an effect on BP. The cohort of prehypertensive and good sleeping subjects is the result of the inclusion and exclusion criteria, which focused on the primary outcome and did not set criteria for sleep quality, which represents a major limitation with respect to the secondary outcome. Finally, although HYP is usually performed continuously over 20–30 min, we used a different, intermittent type of protocol (6 × 5 min with 5-min breaks). We chose to use this protocol in order to accommodate the IH bouts in between the HYP bouts. Also, we do not think that this affected the results since both continuous and intermittent types of aerobic exercise have been shown to induce PEH and improve sleep (Kredlow et al., 2015; Pimenta et al., 2019).

It could be argued that a longer period would be required to detect drops in BP following HYP or HYP&IH. Although existing literature concerning PEH and whole-body exercise suggests extending measurements for up to 120 min (de Brito et al., 2019), it also seems that in most cases, when acute reductions in BP are present these are already manifest 30 min post-exercise (de Brito et al., 2019) (though not always, see Stone et al., 2020). It is possible, nonetheless, that a longer observation period would have been required to detect a more pronounced drop in BP following HYP or HYP&IH.

## Conclusion

One session of intermittent HYP does not lead to an acute reduction in BP, possibly due to the combination of low exercising muscle mass and eventually low intensity. However, mechanisms affecting BP after whole body exercise and RMT might differ, in both short and long-term adaptations. Also, sleep is not improved after HYP in elderly with good sleep. Whether HYP may improve sleep in elderly with sleep complaints or in snorers remains inconclusive and needs further investigation. The addition of IH had no additional benefits on BP or sleep.

## Data Availability Statement

The raw data supporting the conclusions of this article will be made available by the authors, without undue reservation.

## Ethics Statement

The studies involving human participants were reviewed and approved by Kantonale Ethikkommission Zürich. The patients/participants provided their written informed consent to participate in this study.

## Author Contributions

JS and CS conceived this research project. JS, RE, and RO conducted the experiments and analyzed the data. JS drafted the manuscript, which was finalized by JS and CS. All authors read and approved the final version of the manuscript.

## Conflict of Interest

The authors declare that the research was conducted in the absence of any commercial or financial relationships that could be construed as a potential conflict of interest.
